# Upregulation of HMGB1 promotes vascular dysfunction in the soft palate of patients with obstructive sleep apnea via the TLR4/NF‐κB/VEGF pathway

**DOI:** 10.1002/2211-5463.13533

**Published:** 2022-12-30

**Authors:** Tiantian Su, Cong Li, Yu Zhang, Lei Yue, Yuqin Chen, Xiaoqiong Qian, Song Shi

**Affiliations:** ^1^ ENT Department Tongren Hospital, Shanghai Jiao Tong University School of Medicine China

**Keywords:** angiogenesis, HMGB1, obstructive sleep apnea, soft palate, TLR4 pathway

## Abstract

Obstructive sleep apnea (OSA) is characterized by the collapse of the soft palate in the upper airway, resulting in chronic intermittent hypoxia during sleep. Therefore, an understanding of the molecular mechanisms underlying pathophysiological dysfunction of the soft palate in OSA is necessary for the development of new therapeutic strategies. In the present study, we observed that high mobility group protein box 1 (HMGB1) was released by a large infiltration of macrophages in the soft palate of OSA patients. The toll‐like receptor 4/nuclear factor kappa B pathway was observed to be activated by the release of HMGB1, and this was accompanied by an increased expression of pro‐inflammatory factors, including tumor necrosis factor‐α and interleukin‐6. Importantly, increased expression of toll‐like receptor 4 was observed in endothelial cells, contributing to upregulation of the angiogenesis‐related factors vascular endothelial‐derived growth factor and matrix metalloproteinase 9. Moreover, we confirmed the effect of the HMGB1‐mediated toll‐like receptor 4/nuclear factor kappa B pathway on cell proliferation and angiogenesis in an *in vitro* cell model of human umbilical vein endothelial cells. We conclude that HMGB1 may be a potential therapeutic target for preventing angiogenesis and pathology in OSA.

## Graphical abstract

In the soft palate of obstructive sleep apnea patients, high mobility group protein box 1 (HMGB1) was released by a large infiltration of macrophages. The released HMGB1 acts on toll‐like receptor 4 (TLR4) of endothelial cells, then activates the TLR4/nuclear factor kappa B (NF‐κB) pathway and phosphorylates NF‐κB p65. Finally, pro‐inflammatory factors, including tumor necrosis factor‐α and interleukin‐6, and angiogenesis‐related factors, vascular endothelial‐derived growth factor (VEGF) and matrix metalloproteinase 9 (MMP9), are upregulated by the activation of the HMGB1‐mediated TLR4/NF‐κB pathway.

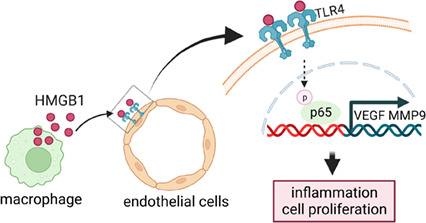

**Abbreviations**
DAPI: 4′,6‐diamidino‐2‐phenylindole
HMGB1: high mobility group protein box 1
HUVECs: human umbilical vein endothelial cells
IH: intermittent hypoxia
IL: interleukin
MMP9: matrix metalloproteinase 9
NF‐κB: nuclear factor kappa B
OSA: obstructive sleep apnea
RAGE: the receptor for advanced glycation end products
TLR4: toll‐like receptor 4
TNF: tumor necrosis factor
VEGF: vascular endothelial‐derived growth factor

Obstructive sleep apnea (OSA) is a widely prevalent health disease, accounting for approximately 9% of middle‐aged men and 4% of the women adult population [1]. This disorder is characterized by the collapse of the soft palate in the upper airway, resulting in chronic intermittent hypoxia (IH) during sleep [2]. Several studies have demonstrated that the collapse of the soft palate might result from pathophysiological changes in inflammatory infiltration, an increase in mucosa thickness and abnormal afferent nerve endings [3]. It was also reported that soft palates from OSA patients were characterized by mucous gland hypertrophy, focal atrophy of muscle fibers and extensive edema of the lamina propria [4]. Our previous study also showed that sensory nerves were damaged and the vascular vessel was enlarged, in addition to caveolin 1 being overexpressed, in chronic IH‐induced soft palate injury rat models [5]. The soft palate has received increased attention because of its involvement with OSA [6, 7]. Therefore, understanding the molecular mechanisms with respect to pathophysiological dysfunction of the soft palate in OSA patients is necessary.

High mobility group protein box 1 (HMGB1) is a nuclear DNA‐binding protein and considered as a damage‐associated molecular pattern [8, 9]. Under normal physiological conditions, HMGB1 proteins are mainly present in the nucleus of examined cells. After cytokine stimulation or hypoxia stress, HMGB1 expression can be transferred from the nucleus to the cytoplasm, followed by release into the extracellular space [10, 11]. These HMGB1 are mainly secreted from necrotic cells, damaged cells or inflammatory cells such as macrophages [12, 13]. Secreted HMGB1 can bind to a variety of cell surface receptors, including advanced glycation end products (RAGEs), Toll‐like receptor (TLR)‐4 and TLR2 [14, 15, 16]. TLR4 is the most studied receptor for endogenous extracellular HMGB1 [13, 15]. Studies have shown that high glucose can induce TLR4 overexpression of human umbilical vein endothelial cells (HUVECs) [17]. Consequently, HMGB1‐mediated TLR4 overexpression could activate the nuclear factor kappa‐B (NF‐κB) signaling pathway [18], thereby triggering a cascade effect. Accordingly, excessive pro‐inflammatory cytokines and chemokines including tumor necrosis factor‐α (TNF‐α), interleukin‐6 (IL‐6) and interleukin‐1β (IL‐1β) are produced to induce inflammation [19, 20, 21]. Exposed to hypoxia, HMGB1 could be secreted by endothelial cells and the upregulation of HMGB1 in turn promotes the proliferation or migration of these endothelial cells [22]. However, the contribution of HMGB1 to vascular endothelial dysfunction of the soft palate in OSA patients is not known.

In the present study, we elucidated the expression and location of HMGB1 in soft palate tissues from OSA patients and investigated the HMGB1‐mediated mechanism for intermittent hypoxia‐induced soft palate injury. Human umbilical vein endothelial cells were used as a cell model to investigate the biological effects and underlying mechanism of HMGB1. We look forward to finding a potential therapeutic target for preventing the angiogenesis and pathology of the soft plate in OSA patients.

## Materials and methods

### Study population

The present study enrolled patients diagnosed with sleep apnea related symptoms from Ear, Nose and Throat Department of Tongren Hospital between January 2021 and July 2021. Inclusion criteria were: subjects who (a) were 18–65 years old and (b) had OSA symptoms including apnea during sleep, snoring and excessive daytime sleepiness. Exclusion criteria were: subjects who had (a) a body mass index > 40 kg·m^−2^; (b) previous oropharyngeal OSA surgery; (c) smoking > 20 years; and (d) a history of critical cardiopulmonary disease, genetic syndrome or neuromuscular disease. To investigated the role of HMGB1 on the soft palate injury of OSA, eight patients (five females and three males; aged 20–50 years) with apnea hypopnea index < 5 events per hour were defined as the control group. In addition, 11 patients (four females and seven males; aged 28–41 years) with apnea hypopnea index > 25 events per hour were defined as the severe OSA group. The study was approved by the bioethical committee of the Tongren Hospital (No. 2021‐001‐01). Written informed consent was obtained from each patient before enrolment in the study. The study methodologies conformed to the standards set by the Declaration of Helsinki. The detailed clinical parameters of the patients are shown in Table 1.

**Table 1:** Patient clinical parameters. BMI, body mass index; AHI, arterial tone apnea/hypopnea index; SaO_2_, arterial oxygen saturation; TST, total sleep time; ESS, Epworth sleepiness scale. Data are the mean ± SD.

	Control group	OSA group
Age (years)	33.38 ± 12.21	34.91 ± 4.93
Gender (female/male) (*n*)	5/3	4/7
BMI (kg·m^−2^)	23.39 ± 3.43	24.75 ± 4.32
AHI (events·h^−1^)	3.54 ± 1.21	47.32 ± 18.69**
SaO_2_ (%)	59.63 ± 7.82	59.27 ± 15.79
Snoring (% of TST)	12.91 ± 4	32.65 ± 7.4**
ESS score	2.59 ± 1.08	11.98 ± 4.04**

** *P* < 0.001 versus control.

### Cell culture and establishment of an *in vitro* model of IH

HUVECs were purchased from the Chinese Academy of Sciences Cell Bank (Shanghai, China). Cells were cultured in endothelial growth medium‐2 (Lonza, Basel, Switzerland) consisting of 5% fetal bovine serum (Gibco, Waltham, MA, USA), 1% streptomycin and penicillin, and supplementation of growth factors in a 37 °C humidified incubator. To obtain an *in vitro* model of IH, HUVECs were exposed to hypoxia (1% O_2_, 5% CO_2_ and 94% N_2_ for 60 min) and then normoxia (21% O_2_, 5% CO_2_ and 74% N_2_ for 30 min) [23]. An oxygen detector was used to continuously monitor oxygen fractions in the chambers. The HUVECs were exposed to IH for six cycles. A control group was exposed to room air for the same period. 5% CO_2_ was employed through the whole cycles. For treatment with HMGB1, recombinant HMGB1 (MCE, HY‐P73102) was added to cultured medium at a final concentration of 1 μg·mL^−1^ and incubated at 37 °C for 48 h.

### Cell viability

A Cell Counting Kit‐8 (CCK‐8; Beyotime, Shanghai, China) was used to measure the cell proliferation of HUVECs. 5 × 10^3^ HUVECs per well were seeded in 96‐well plates. Each group had three duplicate wells. CCK‐8 solution was allowed to interact with the cells at 37 °C for 2 h after different conditional treatments. The absorbance of the colored solution at 450 nm was detected by spectrophotometry. The experiment was repeated three times to ensure data reproducibility.

### Histology and immunohistochemistry

Soft palate tissues were harvested from OSA and control groups, then fixed with paraformaldehyde and embedded in paraffin. For histology, paraffin sections (4 μm thick) were stained with hematoxylin and eosin. For immunohistochemistry, paraffin sections were de‐paraffinized, rehydrated and antigen retrieved with incubation in 0.01 m citrate buffer (pH 6.0) at 121 °C per 100 kpa for 2 min. Slides were incubated with primary antibodies including anti‐CD68 (dilution 1 : 200; ab125212; Abcam, Cambridge, UK), anti‐HMGB1 (dilution 1 : 200; ab18256; Abcam), anti‐TLR4 (dilution 1 : 200; GB11519; Servicebio, Woburn, MA, USA) and anti‐CD31 (dilution 1 : 200; ab28364; Abcam) at 4 °C overnight. Then secondary antibody conjugated with fluorescent dye were incubated at 37 °C for 30 min. Nuclei were stained with 4′,6‐diamidino‐2‐phenylindole (DAPI) (Sigma‐Aldrich, St Louis, MO, USA). Images were acquired with a 50i Nikon fluorescence microscope (Nikon, Tokyo, Japan) and processed with photoshop cs4 software (Adobe Sytems, San Jose, CA, USA).

### RNA isolation, reverse transcriptase PCR and quantitative PCR

Total RNA from soft palate tissues was isolated using Trizol reagent (Invitrogen, Carlsbad, CA, USA). All reverse transcriptase reactions were performed with SuperScript II reverse transcriptase reagent (Invitrogen) in accordance with the manufacturer's instructions. Quantitative PCR was carried out with SYBR Green master mix (Applied Biosystems, Foster, CA, USA) in three repeats of each sample on ABI‐7900 (Applied Biosystems). The relative expression was calculated by the $2^{-\Delta \Delta C_{T}}$ method and normalized by β‐actin. The primer sequence information was: TLR4: Forward 5′‐AAGCCGAAAGGTGATTGTTG‐3′, Reverse 5′‐CTGTCCTCCCACTCCAGGTA‐3′; TLR2: Forward 5′‐CAGCAGGTTCAGGATGTCCG‐3′, Reverse 5′‐AGGTTCTCCACCCAGTAGGC‐3′; RAGE: Forward 5′‐CCACTGGTGCTGAAGTGTAAGG‐3′, Reverse 5′‐GGACTCGGTAGTTGGACTTGGT‐3′.

### ELISA

Serum from OSA patients and control subjects was used to detect HMGB1 level using an ELISA kit (EPX010‐12422‐901; Invitrogen). Tissue homogenate from the soft palate of OSA patients and control subjects was collected. ELISA kits for IL‐6 (ab178013; Abcam) and TNF‐α (ab285312; Abcam) were used to determine IL‐6, TNF‐α levels in accordance with the manufacturer's instructions.

### Western blotting

Total proteins from soft palate tissues were extracted with a Total Protein Extraction Kit (Merck Millipore, Burlington, MA, USA) in accordance with the manufacturer's instructions . Proteins were loaded onto 10% SDS/PAGE and then transferred to poly(vinylidene difluoride) membranes (Merck Millipore). The transferred membranes were blocked in 5% BSA for 1 h at room temperature. Membranes were incubated at 4 °C overnight with primary antibodies against HMGB1 (dilution 1 : 2000; ab18256; Abcam), CD68 (dilution 1 : 1000; ab125212; Abcam), CD31, TLR4 (dilution 1 : 1000; GB11519; Servicebio), p‐NF‐κB p65 (dilution 1 : 2000; 8214S; Cell Signaling Technology, Danvers, MA, USA), VEGF (dilution 1 : 2000; 500661S; Cell Signaling Technology), MMP9 (dilution 1 : 2000; 13667S; Cell Signaling Technology), IL‐6 (dilution 1 : 2000; ab233706; Abcam) and GAPDH (dilution 1 : 10 000; 5174S; Cell Signaling Technology). Appropriate horseradish peroxidase‐conjugated secondary antibodies were incubated at 37 °C for 30 min. Finally, protein bands were detected with a chemiluminescent substrate.

### Statistical analysis

All experiments were performed at least three biological replicates. Data are presented as the mean ± SD and analyzed by Student's *t* test using prism, version 5.0 (GraphPad Software Inc., San Diego, CA, USA). *P* < 0.05 was considered statistically significant.

## Results

### Extensive infiltration of macrophages occurs in the soft palate of patients with OSA

**Fig. 1:**
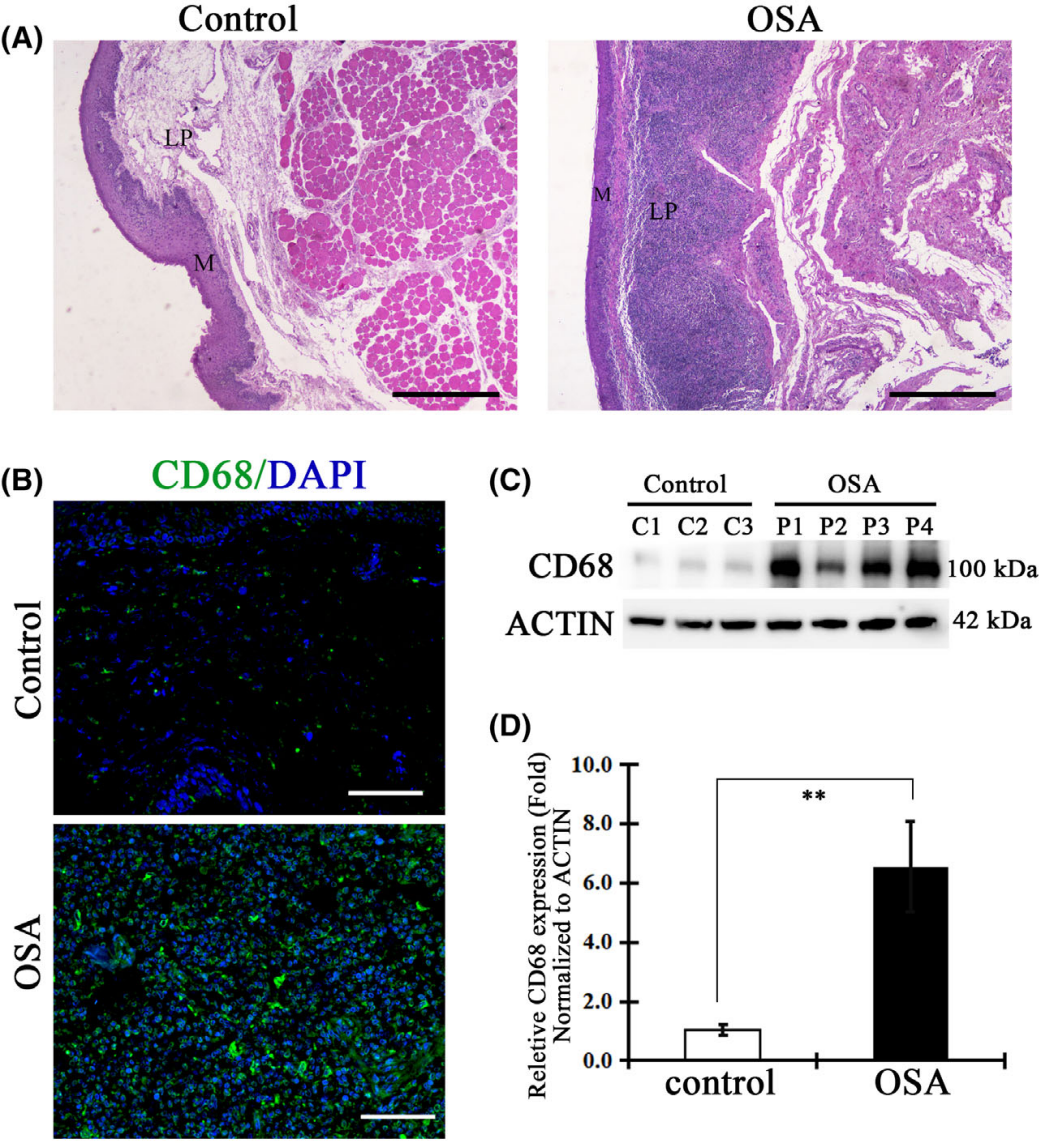
Macrophages were infiltrated into the lamina propria of the soft palate in OSA patients. (A) Morphological structure of the mucosa, lamina propria and muscle layer in the soft palate from control and OSA patients was analyzed by hematoxylin and eosin staining (*n* = 3/control, *n* = 4/OSA). Scale bar = 1 mm. M, mucosa; LP, lamina propria. (B) Immunohistochemistry staining of CD68 in soft palate tissues. Extensive CD68 positive cells infiltrated into the lamina propria of the soft palate in OSA patients (*n* = 3/control, *n* = 4/OSA). Scale bar = 100 μm. (C) The protein expression of CD68 in the soft palate from control and OSA patients was detected by western blotting. C1–3 and P1–4, respectively, present samples from different control subjects and OSA patients. (D) The bar graph indicates the quantitation of protein levels. The fold change of the CD68 level was calculated based on control subjects considered as equal to 1. Data are expressed as the mean ± SD. **P < 0.01 versus control group. *P* values were analyzed by a two‐tailed Student's *t* test.

The inflammatory pathway has been suggested to be activated by IH of OSA, which was confirmed to be a primary reason for vascular endothelial injury [24]. First, the assessment of inflammatory cell infiltration between the control group and OSA patients revealed a significant difference. On hematoxylin and eosin staining, a large number of inflammatory cells were infiltrated into the submucosal layer and lamina propria of the soft plate from patients with OSA, whereas there were only a few inflammatory cells in the control subjects (Fig. 1A). This indicated that inflammation might be involved in the soft palate of OSA patients. Macrophages have been reported as the predominant participants in inflammatory responses. To confirm the role of macrophages in hypoxia‐induced inflammation infiltration, we examined the expression and location of macrophage in the soft palate with the surface marker CD68. Immunohistochemical staining in the soft palate sections indicted a significant increase of CD68 positive cells in the lamina propria of the OSA group (Fig. 1B). In addition, the expression level of CD68 was approximately seven‐fold higher in OSA patients compared to that in control individuals (Fig. 1C,D). These results demonstrated that, in OSA patients, IH would induce extensive infiltration of macrophages in the soft palate.

### Infiltrated macrophages of OSA patients express, translocate and release HMGB1 from the nucleus to the extracellular milieu

**Fig. 2:**
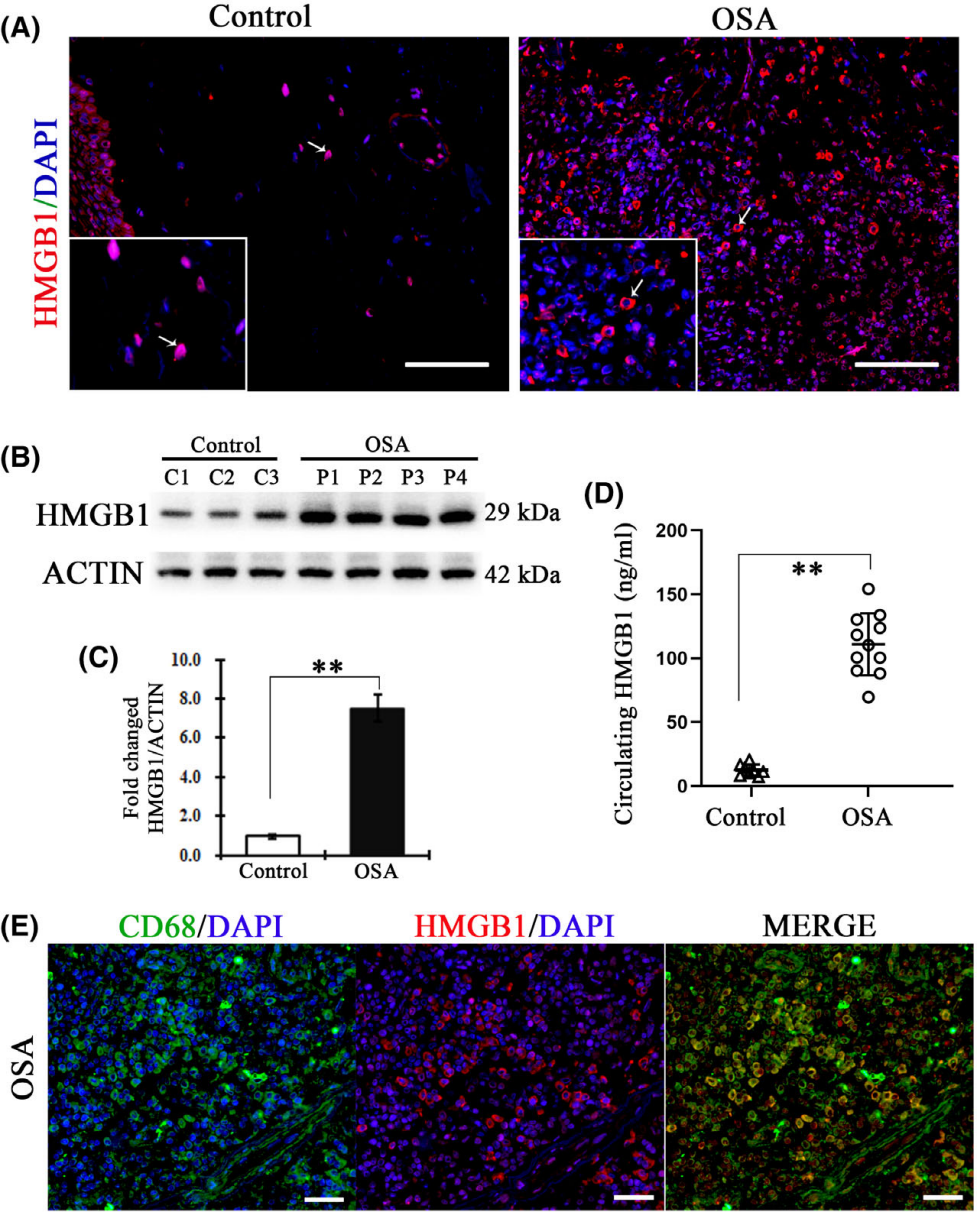
Extensive HMGB1 was secreted by macrophages and translocated from the nucleus to the cellular plasma. (A) Immunohistochemistry staining of HMGB1 in soft palate tissues. Left: an arrow indicates the nucleus of cells in normal control soft palate. Right: an arrow indicates the cellular plasma in the soft palate of OSA patients. Box: a magnified view. (B) The protein expression of HMGB1in soft palate from control and OSA patients was determined by western blotting. C1–3 and P1–4, respectively, present samples from different control subjects and OSA patients. (C) Quantitative analysis results. Data are expressed as the mean ± SD. **P < 0.01 versus control group. *P* values were analyzed by a two‐tailed Student's *t* test. (D) Circulating HMGB1 was measured in serum from healthy controls or OSA patients. A triangle indicates the levels of control subjects (*n* = 8). Open circles indicate the levels of individual patients (*n* = 11). The line and error bars show the mean and 95% confidence intervals. **P < 0.01 versus control group. *P* values were analyzed by atwo‐tailed Student's *t* test. (E) Localization of HMGB1 in the soft palate sections was detected by co‐immunohistochemistry with HMGB1 and CD68. Left: co‐staining of CD68 (green) and DAPI (blue). The green surrounds the blue, which represents the cellular nucleus. The middle image shows co‐staining of HMGB1 (red) and DAPI (blue). Right: merge of green and red. Scale bar = 100 μm.

To identify a role for HMGB1 in the inflammatory response, we examined the HMGB1 level in soft palate tissues and serum from OSA patients and control patients. Immunohistochemical analysis showed only a few HMGB1 positive cells in the submucosal layer and lamina propria of the normal soft plate and these HMGB1 were mainly located in the nucleus of cells (Fig. 2A). However, there were widespread HMGB1 positive cells in the submucosal layer and lamina propria of the soft plate tissues from OSA patients. Importantly, the expression of HMGB1 was transferred to the extranuclear region (cytoplasmic and cell vicinity) of cells (Fig. 2A), suggesting that HMGB1 can translocate from the nucleus to the extracellular milieu. In addition, western blotting demonstrated that the expression of HMGB1 from OSA patients was sharply increased, which was significantly higher (11.73 ± 3.11‐fold) compared to that from normal individuals (0.98 ± 0.11‐fold) (Fig. 2B,C). Furthermore, we measured serum HMGB1 in OSA patients versus healthy controls. The mean serum HMGB1 concentration in the serum of OSA patients was almost ten‐fold higher compared to in healthy controls (110.8 versus 12.5 ng·mL^−1^ HMGB1) (Fig. 2D). To investigate whether the expression of HMGB1 was released by infiltrated macrophages, co‐staining of HMGB1 and CD68 was analyzed. As shown in Fig. 2E, the majority of HMGB1 protein were released from CD68 positive macrophages in the lamina propria of the soft plate tissues from OSA patients. All of these data demonstrated that HMGB1 were highly expressed by infiltrated inflammatory cells and released into the extracellular milieu in OSA patients, which might enhance the collapse of the soft palate.

### The HMGB1‐mediated TLR4/NF‐κB pathway contributes to the inflammatory response in OSA patients

**Fig. 3:**
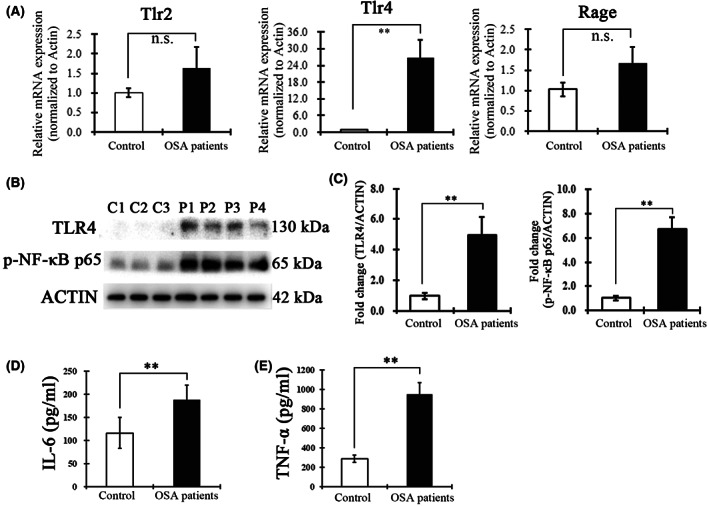
HMGB1‐mediated TLR4/NF‐κB activation and inflammatory factor overexpression in OSA patients. (A) Quantitative PCR detection showing the relative mRNA expression of Tlr2, Tlr4 and Rage in the soft palate from control and OSA patients. (B) Western blot analysis showing the significantly increased TLR4 and p‐NF‐κB p65 levels in OSA patients. C1–3 and P1–4, respectively, present samples from different control subjects and OSA patients. (C) The bar graph indicates the relative expression of TLR4 and p‐NF‐κB p65. The fold change was calculated and compared with control subjects. (D, E) ELISA analysis showing IL‐6 (D) and TNF‐ɑ (E) levels in tissue homogenate of the soft palate from control (*n* = 8) and OSA (*n* = 11) patients. Data are expressed as the mean ± SD. **P < 0.01 versus control group. n.s., no significance. *P* values were analyzed by a two‐tailed Student's *t* test (A, C–E).

To identify the candidate receptor of HMGB1 in the soft plate of OSA patients, we first examined whether TLR2, TLR4 or RAGE was upregulated in response to hypoxia‐induced soft palate injury. RNA isolated from the soft palate of OSA patients and controls was reverse‐transcribed, and TLR2, TLR4 or RAGE messenger RNA was assessed by a quantitative PCR. As shown in Fig. 3A, the TLR4 expression level was approximately 25‐fold higher in the soft palate from OSA patients compared to controls. However, TLR2 and RAGE expression was not statistically different between OSA patients and control individuals. Next, western blot analysis indicated that TLR4 and p‐NF‐κB p65 expression in the soft palate from OSA patients increased significantly compared to of control individuals (Fig. 3B,C), indicating that over expression of HMGB1 could result in binding to TLR4 and activation of the TLR4/NF‐κB pathway. Next, to test the role of NF‐κB activation on transcription and generation of pro‐inflammatory factors, the expression of TNF‐α and IL‐6 in soft palate tissues of healthy and OSA patients was detected. ELISA assays indicated that after activating the TLR4/NF‐κB signal pathway, the expression of TNF‐α and IL‐6 was significantly increased (Fig. 3D,E), which might comprise positive feedback of the inflammatory response. These results suggest that released HMGB1 in the soft plate of OSA patients plays an essential role in TLR4/NF‐κB pathway mediated inflammatory responses.

### HMGB1/TLR4/NF‐κB signaling with endothelial cells drives angiogenesis in the soft palate of OSA patients

**Fig. 4:**
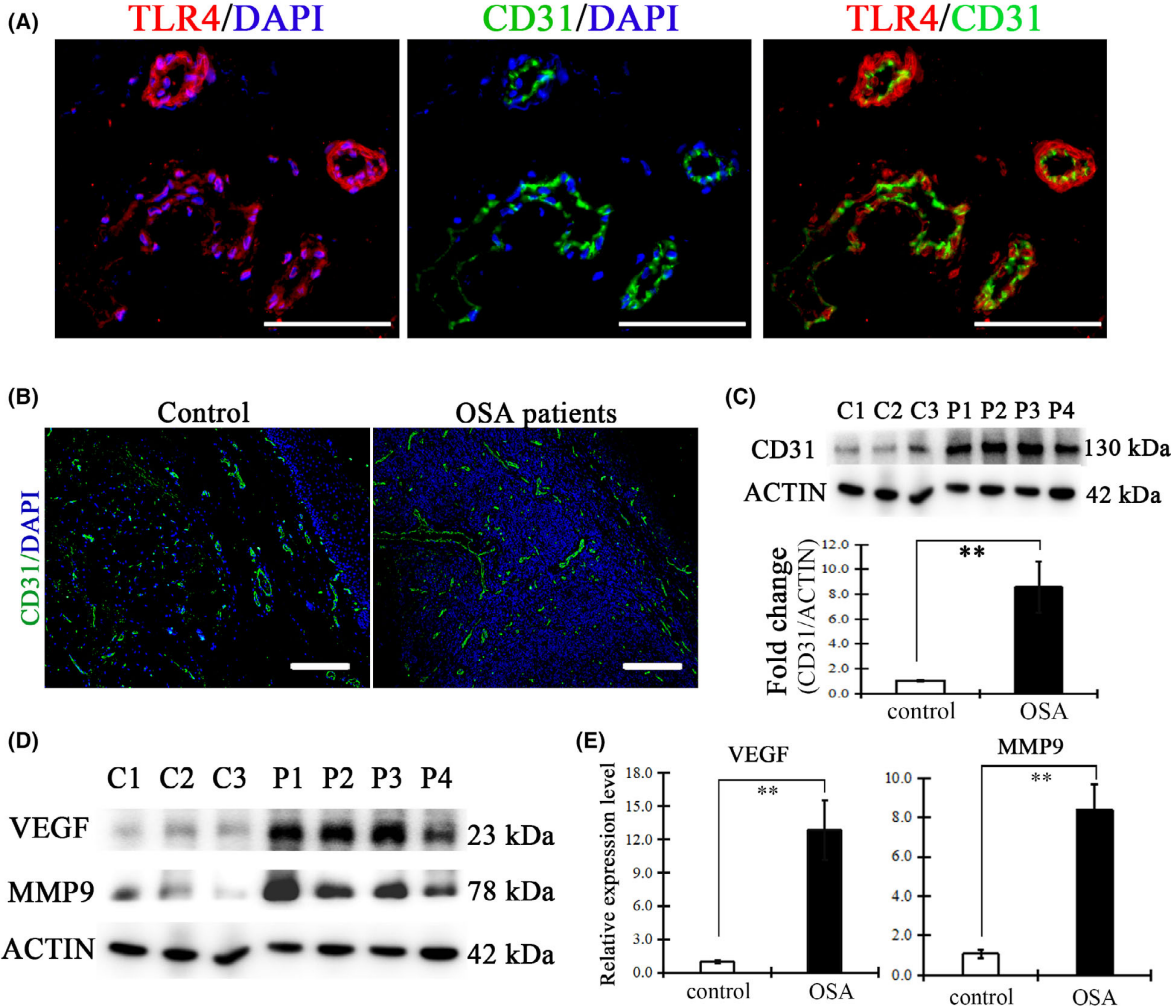
Overexpression of TLR4 in vascular endothelial cells mediated VEGF and MMP9 production. (A) Co‐immunohistochemistry staining with TLR4 and CD31 in soft palate tissues from OSA patients. Red indicates TLR4 positive expression; green indicates CD31 positive cells. (B) Representative image of CD31 staining in the soft palate of control group or OSA group (*n* = 3/control, *n* = 4/OSA). (C) Up blot bands showing western blotting of the CD31 level in the soft palate of control and OSA patients. The down graph inidcates the relative expression of CD31. The fold change was calculated based on control subjects considered as equal to 1. (D) Western blotting showing the expression of VEGF and MMP9 in the soft palate from control and OSA patients. (E) Relative expression of VEGF and MMP9. C1–3 and P1–4, respectively, present samples from different control subjects and OSA patients. The fold change of CD31, VEGF and MMP9 level was calculated based on control subjects considered as equal to 1. Data are expressed as the mean ± SD. **P < 0.01 versus control group. Scale bar = 100 μm. *P* values were analyzed by a two‐tailed Student's *t* test.

Our previous study demonstrated angiogenesis in chronic IH‐induced soft palate damage [5]. We hypothesized that HMGB1‐mediated TLR4 signaling with endothelial cells contributed to angiogenesis. In support of this hypothesis, *in situ* immunohistochemical staining of TLR4 and CD31, a marker for vascular endothelial cell, was performed in the soft palate tissue section of OSA patients. We found the co‐location of TLR4 and CD31 in soft palate tissues (Fig. 4A), suggesting the HMGB1‐mediated TLR4 signaling was activated in CD31 positive endothelial cells of the soft palate. Furthermore, as shown in *in situ* sections, blood vessels were significantly increased in the lamina propria of the soft palate from OSA patients (Fig. 4B). The results of CD31 expression level as analyzed by western blotting indeed confirmed an over‐proliferation of vascular endothelial cells (Fig. 4C). Next, we aimed to directly determine whether the TLR4 pathway upregulated cytokines and angiogenesis matrix metalloproteinases (MMPs) that were linked to angiogenesis. vascular endothelial‐derived growth factor (VEGF) and MMP9, which have been reported to increase in OSA patients [25], were detected by western blotting. As shown in Fig. 4D,E, there was a significant increase of VEGF and MMP9 in the soft palate of OSA patients compared to that in healthy subjects. All of these results suggested that HMGB1 binds to the TLR4 surface marker of endothelial cell and then promotes the production of VEGF and MMP9 via the TLR4/NF‐κB pathway, contributing to angiogenesis of the soft palate from OSA patients.

### Recombinant HMGB1 enhances the proliferation and VEGF production of HUVECs with or without IH treatment via the TLR4/NF‐κB pathway

**Fig. 5:**
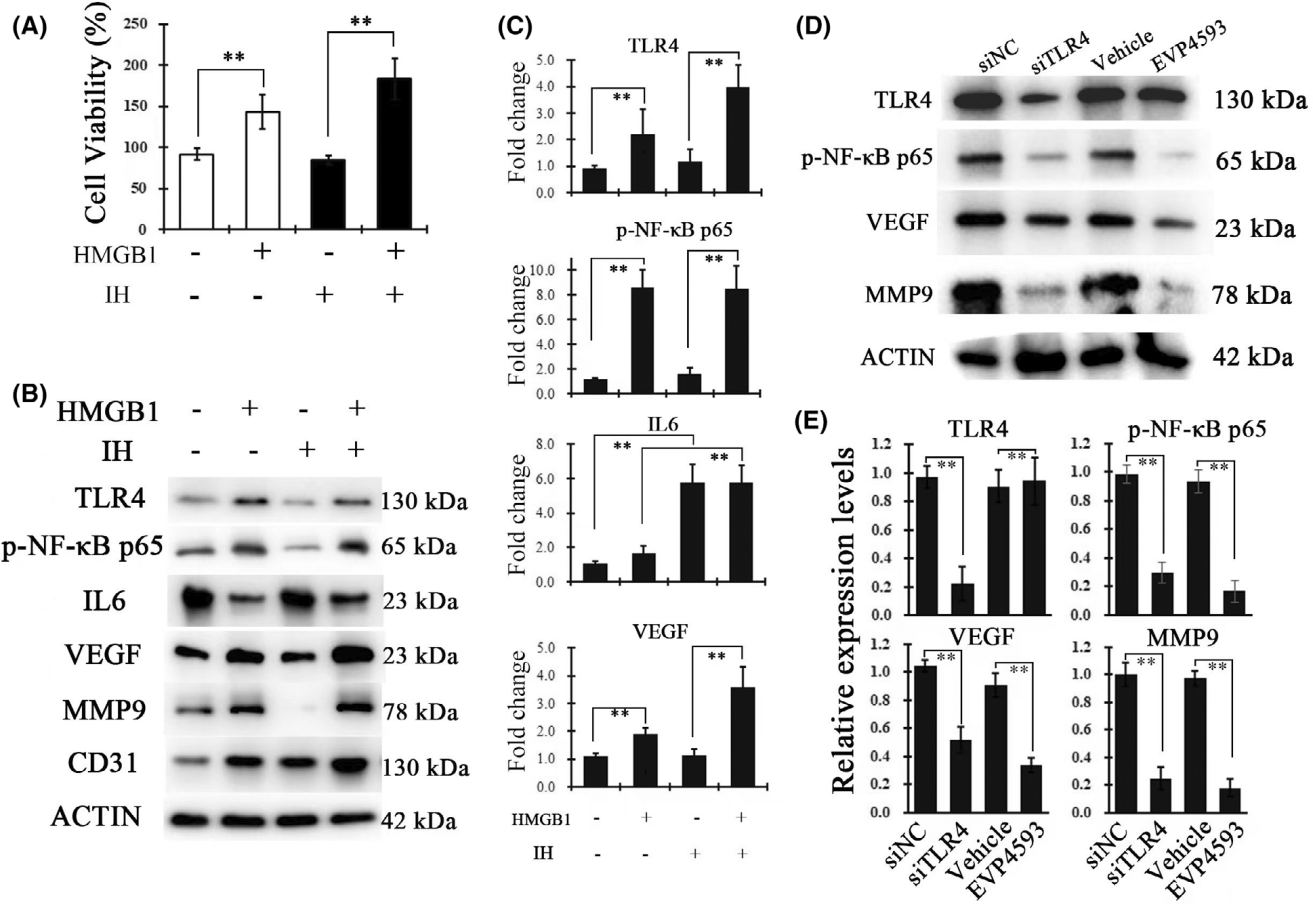
Recombinant HMGB1 enhanced the proliferation and VEGF production of HUVECs via the TLR4/NF‐κB pathway. (A) Cell viability of HUVECs cultured in different conditions was measured using a CCK‐8 (*n* = 3 per group). (B) The protein expression level of TLR4, p‐NF‐κB p65, VEGF, MMP9, IL‐6 and CD31 was measured by western blotting. Cell samples from HUVECs culture under IH or normoxic conditions after rHMGB1 or DMSO treatment (*n* = 3 per group). (C) The fold change of protein levels was calculated as the average expressions normalized to β‐actin. Protein expression in the control group was taken as the baseline and considered equal to 1. (D) Western blotting analysis of TLR4, p‐NF‐κB p65, VEGF and MMP9 levels in rHMGB1 stimulated HUVECs after treating with TLR4 knockdown and NF‐κB inhibitor (EVP4593). (E) The bar graph indicates the relative expression of protein levels normalized to β‐actin. Data are expressed as the mean ± SD. **P < 0.01. *P* values analyzed by a two‐tailed Student's *t* test.

To confirm the HMGB1 mediated TLR4/NF‐κB pathway was the key mechanism for endothelial cell dysfunction of IH‐exposed soft palate, we choose HUVECs as a cell model. Recombinant HMGB1 (rHMGB1) was added in the culture medium when HUVECs were cultured in IH or normoxic conditions. The CCK‐8 results revealed that cell viability was increased significantly after HMGB1 treatment in HUVECs with IH or normoxic conditions (Fig. 5A). Moreover, western blotting indicated that HMGB1 treatment significantly enhanced the protein expression level of TLR4, p‐NF‐κB p65, VEGF and MMP9, whereas IH treatment resulted in no significant difference of these proteins (Fig. 5B,C). However, IH treatment could also increase the expression of IL‐6, which implies that there might be another pathway activated in HUVECs (Fig. 5B,C). To further demonstrate that the HMGB1‐mediated TLR4/NF‐κB pathway contributes to the production of VEGF and MMP9, loss‐of‐function experiments including TLR4 knockdown and NF‐κB inhibitor were performed in HUVECs exposed to rHMGB1. As shown in Fig. 5D,E, a decrease in the TLR4 protein level could significantly suppress phosphorylation of NF‐κB p65 and reduce the expression of VEGF and MMP9. Similar results were also found after treatment with NF‐κB inhibitor (EVP4593). These results confirmed that the HMGB1‐mediated TLR4/NF‐κB pathway was the main mechanism with respect to vascular dysfunction in the soft palate from OSA patients.

## Discussion

The human soft palate is one of key structures involved in repetitive obstruction and breathing interference during sleep [3]. OSA patients with IH mainly result from a pathological progression followed by the collapse of the soft palate. Several histopathological studies have been performed to assess the pathological features of the soft palate from OSA patients using qualitative, quantitative or morphmetric measures. According to some studies, changes in neurogenic components, muscular activation and vascular structure might be account for the soft palate pathology [26, 27]. Effectively alleviating pathological damage of the soft palate will be helpful for preventing the collapse of the soft palate. In the present study, we investigated the pathological features and underlying molecular mechanism of the soft palate from OSA patients and control individuals. We found that, with hypoxia exposure of OSA patients, an extensive infiltration of macrophages occurred in the lamina propria soft palate. Extensive HMGB1 expressed by macrophages was translocated from the nucleus to the cellular plasma and released into the extracellular milieu, implying that HMGB1 was the driving factor for developing a soft palate pathology.

We tested the most likely candidate receptors of HMGB1, such as TLR2, TLR4 and RAGE, and found that TLR4 was the only receptor activated by HMGB1 in the soft palate. More importantly, our data showed that the overexpression of TLR4 receptor was located in vascular endothelial cells but not monocytes or macrophages, implying the HMGB1‐mediated TLR4 pathway regulated the biological function of vascular endothelial cells. HMGB1 as a proinflammatory cytokine has been shown in diseases of angiogenesis [28, 29]. The HMGB1‐mediated TLR4 pathway was shown to result in the activation of NF‐κB, followed induction of the upregulation of adhesion molecules and the production of angiogenic factors in endothelial cells [30, 31]. Consistent with a previous study, we found that the expression of p‐NF‐κB p65, VEGF and MMP9 was significantly increased in OSA patients. Then, to investigate the activation of HGMB1/TLR4/NF‐κB pathway contributing to vascular endothelial cell dysfunction, we used HUVECs as a cell model. The data demonstrated that recombinant HMGB1 could enhanced the proliferation of HUVECs regardless of whether exposed to IH conditions or normoxic conditions. The expression of TLR4, p‐NF‐κB p65 and VEGF proteins was significantly increased after HMGB1 treatment. These results indicated that angiogenesis in the soft palate of OSA patients was activated by the HGMB1‐mediated TLR4/NF‐κB pathway and accompanied by the production of VEGF and MMP9.

Except for the production of angiogenic factors such as VEGF, we also found that the production of inflammatory factors including IL‐6 and TNF‐α was increased. As reported in previous studies, HMGB1 usually exists in the cell nucleus. However, in the case of cell inflammation, it can be upregulated and passively released into the extracellular space by damaged cells [10]. Extracellular HMGB1 is slightly oxidized to bind to the TLR4 receptor with high affinity [32]. In previous studies, HMGB1 interacts with TLR and could activate the NF‐κB signal, thereby activating TNF‐α, IL‐1 and other inflammatory factors [33]. However, HUVECs exposed to rHMGB1 treatment did not promote the expression of IL‐6, which suggested that other forms of receptor‐mediated proinflammation might be activated by HMGB1.

In our previous study, we observed the upregulation of caveolae‐1 in the chronic IH‐induced soft palate rat model. CAV‐1 is the main constituent component of endothelial cells, which regulates endothelial transcytosis [34]. A recent study showed that HMGB1 increased CAV‐1 protein expression and led to enhance endothelial cell permeability [35]. Immunofluorescence analysis and immunoprecipitation analysis indicated the colocalization of CAV‐1 and TLR4 in HUVECs. Interestingly, in the present study, we have detected the expression of CAV1 in HUVECs with or without rHMGB1 stimulation, as well as in TLR4 knockdown HUVECs. The results indicated that HMGB1 can upregulate the expression of CAV1, which is suppressed after inhibiting the expression of TLR4 (data not shown). Therefore, according to present study, it is suggested that the overexpression of CAV‐1 in the chronic IH‐exposed soft palate was activated by the HMGB1/TLR4 pathway.

## Conflict of interest

The authors declare no conflict of interest.

## Author contributions

SS supervised the study. TS and SS designed the project and wrote the manuscript. TS, CL, YZ, LY, XQ and YC performed the experiments. TS and LY collected clinical patients' samples. TS performed data analysis. SS made manuscript revisions.

## Data Availability

The raw data supporting the conclusions of this study are available from the corresponding author upon reasonable request.

## References

[1] AI Lawati NM , Patel SR , Ayas NT . Epidemiology, risk factors, and consequences of obstructive sleep apnea and short sleep duration. Prog Cardiovasc Dis. 2009;51:285–93. 19110130 10.1016/j.pcad.2008.08.001

[2] Patel SR . Obstructive sleep apnea. Ann Intern Med. 2019;171:ITC81–96. 31791057 10.7326/AITC201912030

[3] Mu L , Chen J , Li J , Arnold M , Sobotka S , Nyirenda T , et al. Sensory innervation of the human soft palate. Anat Rec (Hoboken). 2018;301:1861–70. 30079585 10.1002/ar.23864

[4] Paulsen FP , Steven P , Tsokos M , Jungmann K , Müller A , Verse T , et al. Upper airway epithelial structural changes in obstructive sleep‐disordered breathing. Am J Respir Crit Care Med. 2002;166:501–9. 12186828 10.1164/rccm.2109099

[5] Li C , Zhang Y , Chen Y , Su T , Zhao Y , Shi S . Cell‐autonomous autophagy protects against chronic intermittent hypoxia induced sensory nerves and endothelial dysfunction of soft palate. Med Sci Monit. 2020;26:e920878. 32616707 10.12659/MSM.920878 PMC7353292

[6] Kummer AW . Types and causes of velopharyngeal dysfunction. Semin Speech Lang. 2011;32:150–8. 21948641 10.1055/s-0031-1277717

[7] Lee CH , Hong SL , Rhee CS , Kim SW , Kim JW . Analysis of upper airway obstruction by sleep videofluoroscopy in obstructive sleep apnea: a large population‐based study. Laryngoscope. 2012;122:237–41. 21919011 10.1002/lary.22344

[8] Zhang C , Dong H , Chen F , Wang Y , Ma J , Wang G . The HMGB1‐RAGE/TLR‐TNF‐α signaling pathway may contribute to kidney injury induced by hypoxia. Exp Ther Med. 2019;17:17–26. 30651760 10.3892/etm.2018.6932 PMC6307518

[9] Sessa L , Bianchi ME . The evolution of high mobility group box (HMGB) chromatin proteins in multicellular animals. Gene. 2007;387:133–40. 17156942 10.1016/j.gene.2006.08.034

[10] Bauer EM , Shapiro R , Zheng H , Ahmad F , Ishizawar D , Comhair SA , et al. High mobility group box 1 contributes to the pathogenesis of experimental pulmonary hypertension via activation of toll‐like receptor 4. Mol Med. 2013;18:1509–18. 23269975 10.2119/molmed.2012.00283 PMC3576475

[11] Tang D , Billiar TR , Lotze MT . A Janus tale of two active high mobility group box 1 (HMGB1) redox states. Mol Med. 2012;18:1360–2. 23073660 10.2119/molmed.2012.00314 PMC3533642

[12] Magna M , Pisetsky DS . The role of HMGB1 in the pathogenesis of inflammatory and autoimmune diseases. Mol Med. 2014;20:138–46. 24531836 10.2119/molmed.2013.00164 PMC3966993

[13] Yang H , Antoine DJ , Andersson U , Tracey KJ . The many faces of HMGB1: molecular structure‐functional activity in inflammation, apoptosis, and chemotaxis. J Leukoc Biol. 2013;93:865–73. 23446148 10.1189/jlb.1212662 PMC4051189

[14] Kang R , Chen R , Zhang Q , Hou W , Wu S , Cao L , et al. HMGB1 in health and disease. Mol Aspects Med. 2014;40:1–116. 25010388 10.1016/j.mam.2014.05.001 PMC4254084

[15] Olejarz W , Głuszko A , Cyran A , Bednarek‐Rajewska K , Proczka R , Smith DF , et al. TLRs and RAGE are elevated in carotid plaques from patients with moderate‐to‐severe obstructive sleep apnea syndrome. Sleep Breath. 2020;24:1573–80. 32076951 10.1007/s11325-020-02029-w PMC7679342

[16] Liu Y , Yan W , Tohme S , Chen M , Fu Y , Tian D , et al. Hypoxia induced HMGB1 and mitochondrial DNA interactions mediate tumor growth in hepatocellular carcinoma through toll‐like receptor 9. J Hepatol. 2015;63:114–21. 25681553 10.1016/j.jhep.2015.02.009 PMC4475488

[17] Guo X , Shi Y , Du P , Wang J , Han Y , Sun B , et al. HMGB1/TLR4 promotes apoptosis and reduces autophagy of hippocampal neurons in diabetes combined with OSA. Life Sci. 2019;239:117020. 31678553 10.1016/j.lfs.2019.117020

[18] Palumbo R , Galvez BG , Pusterla T , De Marchis F , Cossu G , Marcu KB , et al. Cells migrating to sites of tissue damage in response to the danger signal HMGB1 require NF‐kappa B activation. J Cell Biol. 2007;179:33–40. 17923528 10.1083/jcb.200704015 PMC2064729

[19] Scaffidi P , Misteli T , Bianchi ME . Release of chromatin protein HMGB1 by necrotic cells triggers inflammation. Nature. 2002;418:191–5. 12110890 10.1038/nature00858

[20] Liu X , Lu B , Fu J , Zhu X , Song E , Song Y . Amorphous silica nanoparticles induce inflammation via activation of NLRP3 inflammasome and HMGB1/TLR4/MYD88/NF‐kb signaling pathway in HUVEC cells. J Hazard Mater. 2021;404:124050. 33053467 10.1016/j.jhazmat.2020.124050

[21] Meng L , Li L , Lu S , Li K , Su Z , Wang Y , et al. The protective effect of dexmedetomidine on LPS‐induced acute lung injury through the HMGB1‐mediated TLR4/NF‐κB and PI3K/Akt/mTOR pathways. Mol Immunol. 2018;94:7–17. 29241031 10.1016/j.molimm.2017.12.008

[22] van Beijnum JR , Nowak‐Sliwinska P , van den Boezem E , Hautvast P , Buurman WA , Griffioen AW . Tumor angiogenesis is enforced by autocrine regulation of high‐mobility group box 1. Oncogene. 2013;32:363–74. 22391561 10.1038/onc.2012.49

[23] Toffoli S , Roegiers A , Feron O , Van Steenbrugge M , Ninane N , Raes M , et al. Intermittent hypoxia is an angiogenic inducer for endothelial cells: role of HIF‐1. Angiogenesis. 2009;12:47–67. 19184477 10.1007/s10456-009-9131-y

[24] Ciccone MM , Scicchitano P , Mitacchione G , Zito A , Gesualdo M , Caputo P , et al. Is there a correlation between OSAS duration/severity and carotid intima‐media thickness? Respir Med. 2012;106:740–6. 22317765 10.1016/j.rmed.2011.12.016

[25] Chuang LP , Chen NH , Lin SW , Chang YL , Chao IJ , Pang JH . Increased matrix metalloproteinases‐9 after sleep in plasma and in monocytes of obstructive sleep apnea patients. Life Sci. 2013;93:220–5. 23792205 10.1016/j.lfs.2013.06.009

[26] Patel JA , Ray BJ , Fernandez‐Salvador C , Gouveia C , Zaghi S , Camacho M . Neuromuscular function of the soft palate and uvula in snoring and obstructive sleep apnea: a systematic review. Am J Otolaryngol. 2018;39:327–37. 29525140 10.1016/j.amjoto.2018.03.006

[27] Liu H , Prot VE , Skallerud BH . Soft palate muscle activation: a modeling approach for improved understanding of obstructive sleep apnea. Biomech Model Mechanobiol. 2019;18:531–46. 30511264 10.1007/s10237-018-1100-1

[28] Tohme S , Yazdani HO , Liu Y , Loughran P , van der Windt DJ , Huang H , et al. Hypoxia mediates mitochondrial biogenesis in hepatocellular carcinoma to promote tumor growth through HMGB1 and TLR9 interaction. Hepatology. 2017;66:182–97. 28370295 10.1002/hep.29184 PMC5481489

[29] De Leo F , Quilici G , Tirone M , De Marchis F , Mannella V , Zucchelli C , et al. Diflunisal targets the HMGB1/CXCL12 heterocomplex and blocks immune cell recruitment. EMBO Rep. 2019;20:e47788. 31418171 10.15252/embr.201947788 PMC6776901

[30] Xu J , Benabou K , Cui X , Madia M , Tzeng E , Billiar T , et al. TLR4 deters perfusion recovery and upregulates toll‐like receptor 2 (TLR2) in ischemic skeletal muscle and endothelial cells. Mol Med. 2015;21:605–15. 26181630 10.2119/molmed.2014.00260 PMC4656200

[31] Lan J , Luo H , Wu R , Wang J , Zhou B , Zhang Y , et al. Internalization of HMGB1 (high mobility group box 1) promotes angiogenesis in endothelial cells. Arterioscler Thromb Vasc Biol. 2020;40:2922–40. 32998518 10.1161/ATVBAHA.120.315151

[32] Sacks D , Baxter B , Campbell BCV , Carpenter JS , Cognard C , Dippel D , et al. Multisociety consensus quality improvement revised consensus statement for endovascular therapy of acute ischemic stroke. Int J Stroke. 2018;13:612–32. 29786478 10.1177/1747493018778713

[33] Kang N , Hai Y , Yang J , Liang F , Gao CJ . Hyperbaric oxygen intervention reduces secondary spinal cord injury in rats via regulation of HMGB1/TLR4/NF‐κB signaling pathway. Int J Clin Exp Pathol. 2015;8:1141–53. 25973000 PMC4396250

[34] Tian XF , Xia XB , Xu HZ , Xiong SQ , Jiang J . Caveolin‐1 expression regulates blood‐retinal barrier permeability and retinal neovascularization in oxygen‐induced retinopathy. Clin Exp Ophthalmol. 2012;40:e58–66. 21794046 10.1111/j.1442-9071.2011.02656.x

[35] Jiang R , Cai J , Zhu Z , Chen D , Wang J , Wang Q , et al. Hypoxic trophoblast HMGB1 induces endothelial cell hyperpermeability via the TRL‐4/caveolin‐1 pathway. J Immunol. 2014;193:5000–12. 25339669 10.4049/jimmunol.1303445

